# Muscle fibrosis as a prognostic biomarker in facioscapulohumeral muscular dystrophy: a retrospective cohort study

**DOI:** 10.1186/s40478-023-01660-4

**Published:** 2023-10-17

**Authors:** Elvira Ragozzino, Sara Bortolani, Lorena Di Pietro, Andrea Papait, Ornella Parolini, Mauro Monforte, Giorgio Tasca, Enzo Ricci

**Affiliations:** 1 Dipartimento Scienze della Vita e Sanità Pubblica, Università Cattolica del Sacro Cuore, Rome, Italy; 2 Unità Operativa Complessa di Neurologia, Fondazione Policlinico Universitario A. Gemelli IRCCS, Rome, Italy; 3 Fondazione Policlinico Universitario A. Gemelli IRCCS, Rome, Italy; 4 Istituto di Neurologia, Università Cattolica del Sacro Cuore, Rome, Italy; 5 John Walton Muscular Dystrophy Research Centre, Newcastle University and Newcastle Hospitals NHS Foundation Trusts, Newcastle Upon Tyne, UK

**Keywords:** Muscle fibrosis, Skeletal muscle, Facioscapulohumeral muscular dystrophy, Neuromuscular disease, Biomarker, Muscle magnetic resonance imaging, Immune cell infiltrates, Muscle degeneration

## Abstract

Facioscapulohumeral muscular dystrophy (FSHD) is an autosomal dominant epigenetic disorder with highly variable muscle involvement and disease progression. Ongoing clinical trials, aimed at counteracting muscle degeneration and disease progression in FSHD patients, increase the need for reliable biomarkers. Muscle magnetic resonance imaging (MRI) studies showed that the appearance of STIR-positive (STIR+) lesions in FSHD muscles represents an initial stage of muscle damage, preceding irreversible adipose changes. Our study aimed to investigate fibrosis, a parameter of muscle degeneration undetectable by MRI, in relation to disease activity and progression of FSHD muscles. We histologically evaluated collagen in FSHD1 patients’ (STIR+ n = 27, STIR− n = 28) and healthy volunteers’ (n = 12) muscles by picrosirius red staining. All patients (n = 55) performed muscle MRI before biopsy, 45 patients also after 1 year and 36 patients also after 2 years. Fat content (T1 signal) and oedema/inflammation (STIR signal) were evaluated at baseline and at 1- and 2-year MRI follow-up. STIR+ muscles showed significantly higher collagen compared to both STIR− (*p* = 0.001) and healthy muscles (*p* < 0.0001). STIR− muscles showed a higher collagen content compared to healthy muscles (*p* = 0.0194). FSHD muscles with a worsening in fatty infiltration during 1- (*P* = 0.007) and 2-year (*P* < 0.0001) MRI follow-up showed a collagen content of 3.6- and 3.7-fold higher compared to FSHD muscles with no sign of progression. Moreover, the fibrosis was significantly higher in STIR+ muscles who showed a worsening in fatty infiltration in a timeframe of 2 years compared to both STIR− (*P* = 0.0006) and STIR+ muscles with no sign of progression (*P* = 0.02). Fibrosis is a sign of muscle degeneration undetectable at MRI never deeply investigated in FSHD patients. Our data show that 23/27 of STIR+ and 12/28 STIR− muscles have a higher amount of collagen deposition compared to healthy muscles. Fibrosis is higher in FSHD muscles with a worsening in fatty infiltration thus suggesting that its evaluation with innovative non-invasive techniques could be a candidate prognostic biomarker for FSHD, to be used to stratify patients and to evaluate the efficacy of therapeutic treatments.

**Supplementary Information:**

The online version contains supplementary material available at 10.1186/s40478-023-01660-4.

## Introduction

Facioscapulohumeral muscular dystrophy (FSHD) is the second most prevalent muscular dystrophy in the adult population, and affects approximately 870,000 people worldwide [1]. The most common form of the disease is type 1 (FSHD1), which is associated with a contraction of the D4Z4 macrosatellite repeat on the subtelomeric region of chromosome 4q35. This shortening, combined with a permissive allele, allows the inappropriate transcription of the *double homeobox 4* (*DUX4*) gene, considered the primary drive for muscle degeneration in patients [2]. FSHD is a slowly progressive disease with an autosomal dominant inheritance, characterized by an extreme variability in age at onset and clinical severity, even in the same family, and by an unpredictable rate of disease progression in different muscles of a single individual [3, 4]. Approximately 20% of patients’ relatives remain asymptomatic although sharing the same FSHD-causing genetic background of the affected probands [3]. Muscle magnetic resonance imaging (MRI) has become the “gold-standard” technique to evaluate and follow muscle involvement and degeneration in FSHD patients [5, 6]. It is a non-invasive method able to detect fatty infiltration, which is a sign of terminal muscle degeneration, on T1 weighted (T1w) sequences, and oedema, which is a sign of inflammation, on short tau inversion recovery (STIR) sequences [7]. MRI studies in FSHD patients showed that muscle degeneration is an asynchronous process as apparently unaffected muscles coexist with affected muscles thus showing various degrees of fatty replacement in the same patient. Recent longitudinal studies pointed out that the appearance of STIR-positive (STIR+) lesions represents an initial stage of muscle damage, preceding and accelerating irreversible adipose changes [8, 9]. Therefore, STIR+ lesions are considered a biomarker of an active phase of the disease characterized by inflammation, faster progression towards fatty replacement, and higher likelihood to express *DUX4* targets [9–13]. There is a wealth of literature about the interrelation among inflammation, the impairment of non-myogenic mesenchymal stromal cells and fibrosis in driving muscle degeneration in muscular disorders [14–17]. In FSHD muscles, the histological identification and characterization of the inflammatory changes dates back to 1995, and both initial and subsequent pathology studies showed the presence of intramuscular T and B lymphocytes [18–20]. Our group has recently demonstrated impaired in vitro properties of non-myogenic mesenchymal stromal cells (historically called fibroadipogenic progenitors) isolated from FSHD patients’ muscles [21]. These cells accumulate in FSHD STIR+ muscles and this expansion correlate to the progressive fibrous-fatty replacement of muscles [21]. These data were in agreement with the results obtained in a FSHD mouse model [22], suggesting a possible involvement of non-myogenic mesenchymal stromal cells in the pathogenesis of the disease. However, detailed studies investigating muscle fibrosis, another feature of dystrophic muscle degeneration, in FSHD patients are lacking. Precisely, collagen deposition was quantified and found increased in muscles of an inducible-*DUX4* mouse model [22] and qualitatively described as a part of FSHD patients’ muscle myophatic changes [12, 20, 23]. Notably, current available MRI scanning protocols are unable to confidently assess muscular collagen deposition [7]. In our study, we aimed at investigating fibrosis in a large cohort of FSHD patients’ muscles, in relation to their radiological features, and to evaluate whether it could be a predictive marker of disease progression. Our data showed that 23/27 STIR+ and 12/28 STIR− FSHD muscles had increased collagen content compared to control muscles. In addition, the amount of fibrosis positively correlated with the lymphocyte infiltration and the progression in fatty replacement, thus distinguishing between progressive and non-progressive FSHD STIR+ muscles in a timeframe of two years.

## Materials and methods

### Patient enrollment and sample collection

We retrospectively analyzed muscle specimens from 55 genetically confirmed FSHD1 patients and 12 healthy controls (Additional file 1: Table S1). Controls were healthy volunteers, including unaffected relatives of FSHD patients, that were age-matched [mean age controls ± standard deviation (SD): 46 ± 16 years, mean age FSHD patients ± SD: 48 ± 14 years], and no past and current history of systemic inflammatory disease (Additional file 1: Table S1). All patients underwent muscle MRI before biopsy, according to previously published protocol [24]. Biopsy procedure was MRI-informed to include muscles with either normal (T1−) or hyperintense (T1+) signals on T1w sequences and muscles with either normal (STIR−) or hyperintense (STIR+) signals on STIR-sequences. A second and third muscle MRI, performed 12 and 24 months after biopsy, were available in 45 and 36 FSHD patients, respectively. Clinical severity was assessed at baseline by the 10-grade clinical severity scale (CSS) [25]. Conchotome muscle biopsy [26] was performed within 2 weeks from the first MRI scan after obtaining signed informed consent. Two neurologists with experience in muscle imaging (E. Ricci; S.B.) assessed all the scans independently. Baseline and follow-up T1w sequences were first evaluated to assign a semi-quantitative score of fatty infiltration of single muscles (named T1-score) using a 5-point semi-quantitative scale (0 = normal appearance; 1 = traces of increased signal intensity; 2 = increased signal intensity with beginning confluence in less than 50% of the muscle; 3 increased signal intensity in more than 50% of an examined muscle; 4 = the entire muscle is replaced by increased signal intensity) [27]. To detect more subtle changes not identified by the former assessment, the observers performed a direct visual comparison between the baseline and the follow-up scan of each patient on the same computer monitor as previously described [9]. Radiological progression in single muscles was defined as an increase in fatty replacement.

### Skeletal muscle sections

Transverse skeletal muscle cryosections of healthy, FSHD STIR+ and FSHD STIR− muscles were obtained from fresh frozen muscle biopsies mounted in optimal cutting temperature compound (OCT 4583, Tissue-Tek®, Sakura) and frozen in isopentane (106056, Merck Millipore) cooled in liquid nitrogen. Seven μm sections were cut with a cryostat at − 25 °C and 2 different sections were processed in duplicates for each patient. The slides with muscle cryosections of the 3 aforementioned groups were used for picrosirius red staining. An immunohistochemistry analysis of inflammation was performed according to the amount of muscle specimens.

### Picrosirius red staining

Collagen detection was performed using 0.1% picrosirius red solution (Sigma Direct Red 80 365548, Sigma Picric Acid Solution P6744) which stains collagens type I and III in red and muscle fibers in pale yellow. Slides were fixed in 4% paraformaldehyde (Sigma) for 10 min, washed twice in tap water for 5 min, and incubated for 1 h with 0.1% picrosirius red solution. Then, the slides were washed 3 times in water with 0.5% acetic acid (64197, J.T. Baker) for 5 min, dehydrated in ethanol (64175, VWR chemicals) and fixed in xylene (1330207, VWR chemicals). Coverslips were mounted with Eukitt mounting medium (SIC). Stained muscle sections were observed under the optical microscope Olympus BH-2 (Olympus Life Science) and non-overlapping 20× images covering the entire surface of each section were acquired with the software Jenoptik-Gryphax V1.1.10.6. The evaluation of fibrosis was performed with ImageJ software (1.53 version) by measuring the total collagen deposition (red) between the muscle fibers (soft yellow) with color subtraction and reported in percentage area [% of muscle occupied by collagen/field].

### Immunohistochemistry

The slides were fixed in cold acetone (VWR chemicals) for 10 min and then washed 3 times in 1× Phosphate Buffered Saline (PBS, Gibco) for 5 min. The slides were incubated for 1 h at room temperature in 1× PBS with 5% of BSA (Bovine Serum Albumin, Sigma) blocking solution. The specific primary antibody [1:100 CD8 (Dako M7103), 1:500 CD4 (Dako M7310), 1:500 CD20 (Dako M0755), 1:50 BDCA1 (Clone AD5-8E7, Miltenyi Biotec)] was incubated overnight at 4 °C in PBS/1% BSA. After 3 washes in 1X PBS for 5 min, horseradish peroxidase-conjugated secondary antibody (MP-7405-15, VectorLaboratories) was added for 1 h at room temperature. Diaminobenzidine (Sigma) was used to reveal the secondary antibody. The slides were washed in tap and distilled water before being dehydrated in ethanol (64175, VWR chemicals), fixed in xylene (1330207, VWR chemicals) and coverslips were mounted with Eukitt mounting medium (SIC). Stained muscle sections were observed under an optical microscope Olympus BH-2 (Olympus Life Science) and 10× non-overlapping images covering the entire surface of each section were acquired with the software Jenoptik-Gryphax V1.1.10.6. The count of immune cell infiltrates (total, endomysial and perivascular immune cells) was performed on the aforementioned images, normalized per mm^2^ of analyzed surface area and reported as number of immune cells/mm^2^ of surface. The total count immune cell infiltrate was calculated only for the muscle specimens analyzed for the presence of all the listed immune markers.

### Statistical analysis

Data were analyzed using GraphPad Prism software (8.4 version; San Diego, CA, USA) and were presented as mean ± SD. Statistical significance level was set for *p* values < 0.05. After checking for normality by the D’Agostino-Pearson omnibus test, differences in variables between groups were analyzed using Mann–Whitney test or Kruskal–Wallis test with Dunn’s post-hoc analysis. Correlations were estimated by Pearson’s correlation test.

## Results

### Fibrosis in FSHD patients’ muscles

**Fig. 1:**
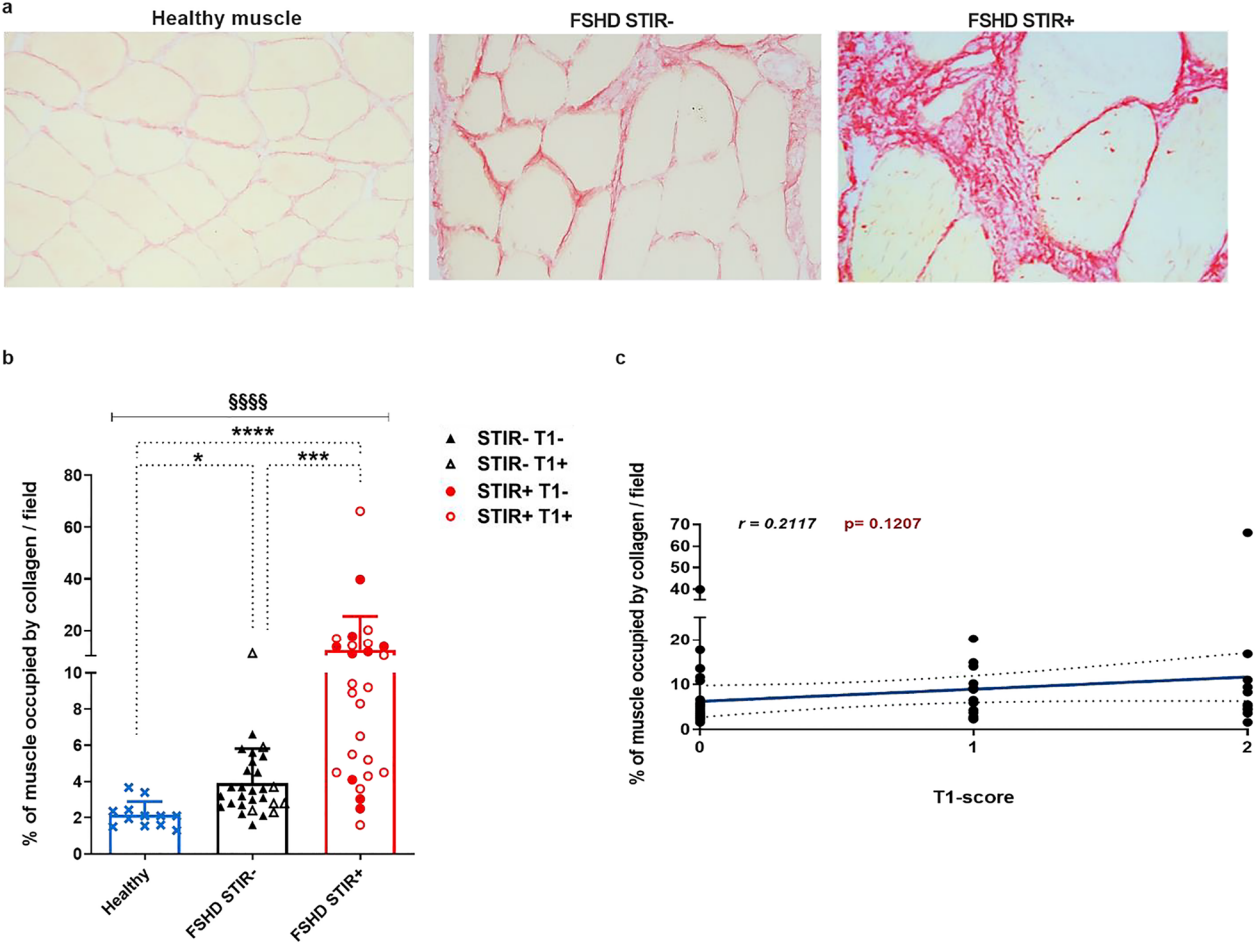
Collagen content of healthy and FSHD muscles.** a** Representative pictures of picrosirius red staining in healthy, FSHD STIR− and FSHD STIR+ muscle sections (20× magnification). **b** The graph shows the percentage of fibrosis per field in healthy (n = 12), FSHD STIR− (n = 28) and FSHD STIR+ (n = 27) muscles. The results are reported as mean ± SD and were compared using Kruskal–Wallis test (§§§§*p* < 0.0001) followed by Dunn’s test (*p < 0.05, ***p < 0.001, ****p < 0.0001). **c** Pearson correlation between the percentage of fibrosis and the MRI T1-score of sampled FSHD muscles

In order to obtain a comprehensive characterization of fibrosis in the context of dystrophic degeneration, we employed picrosirius red staining to quantify the deposition of collagen on muscle sections obtained from MRI-informed muscle biopsies in a cohort of 55 FSHD patients. Specimens were obtained from 27 muscles with (STIR+) and from 28 muscles without (STIR−) signs of disease activity. Data were compared to muscle sections obtained from 12 age-matched healthy volunteers. Information regarding patients, healthy controls, and sampled muscles were summarized in Additional file 1: Table S1. Collagen was significantly higher in FSHD muscles compared to control muscles (Fig. 1a, b), with FSHD STIR+ muscles showing the greatest amount of collagen deposition (Fig. 1a, b). FSHD muscles with (T1+) and without (T1−) MRI features of fatty infiltration did not show significant differences in the amount of intramuscular fibrosis (Fig. 1b), and the amount of fibrosis did not correlate with the T1-score of FSHD muscles at baseline (Fig. 1c). Considering the mean ± 2 standard deviation (SD) of collagen measured in healthy muscles as the upper normal limit threshold (3.6% of collagen/field), 85.2% (23/27) of FSHD STIR+ and 42.9% (12/28) of FSHD STIR− muscles displayed a higher-than-normal extent of collagen accumulation as index of fibrotic muscle degeneration (Table 1).

**Table 1:** Muscles with collagen content over the upper normal limit

Category of muscles	Muscles with fibrosis	% of muscles with fibrosis	Mean of collagen (%)/field of muscles with fibrosis
Healthy	0/12	0.0	0.0
FSHD STIR−	12/28	42.9	5.5
FSHD STIR+	23/27	85.2	13.9

### Composition of immune cell infiltrates in FSHD muscles

**Fig. 2:**
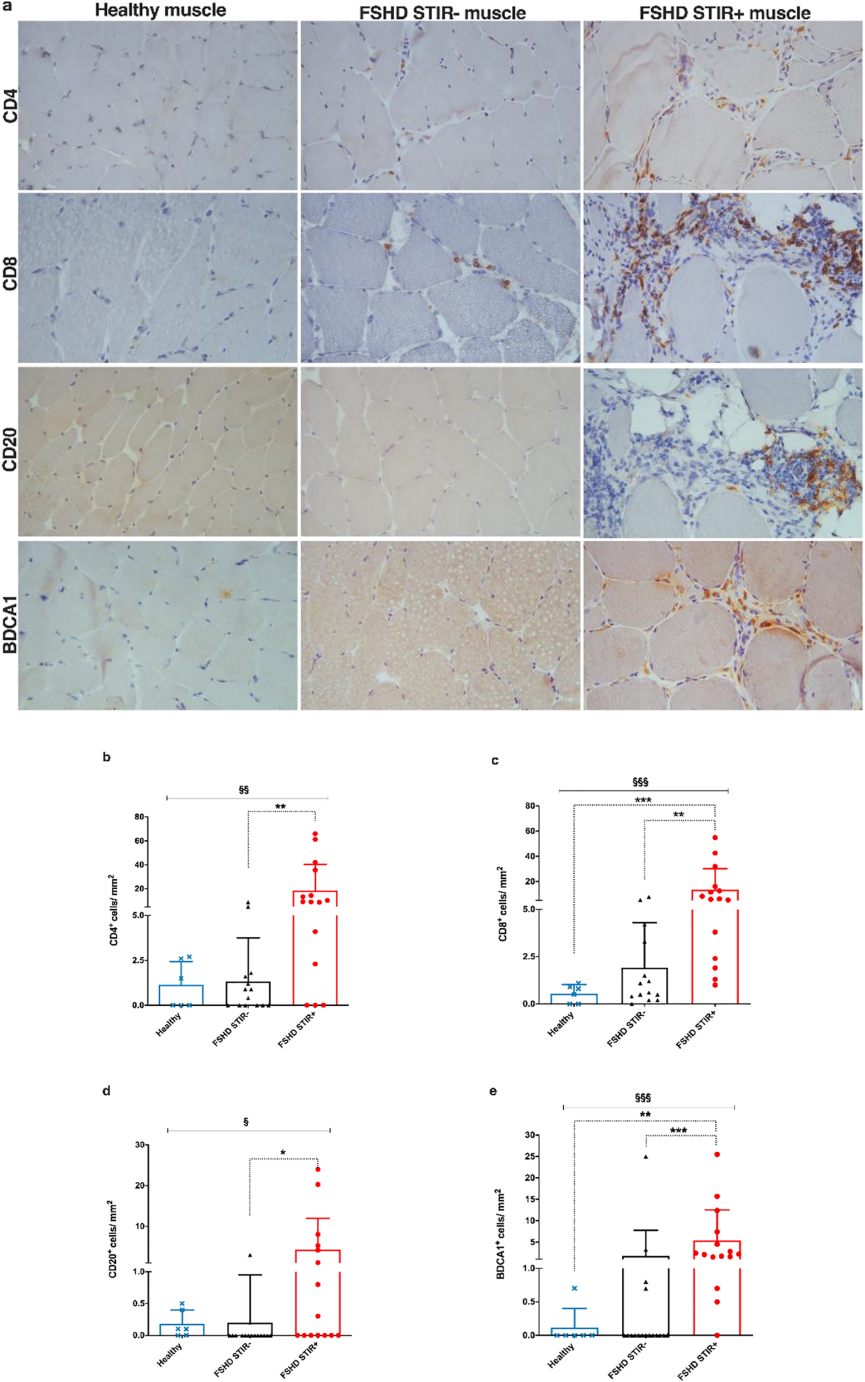
Evaluation of immune cell infiltrates in healthy and FSHD muscles. **a** Representative pictures of peroxidase staining of CD4^+^, CD8^+^, CD20^+^ and BDCA1^+^ cells in healthy, FSHD STIR− and FSHD STIR+ muscle sections (20× magnification). The graphs show the total distribution of CD4^+^ cells/mm^2^ (**b**) in healthy (n = 6), FSHD STIR− (n = 16) and FSHD STIR+ (n = 15) muscles, CD8^+^ cells/mm^2^ (**c**) in healthy (n = 6), FSHD STIR− (n = 14) and FSHD STIR+ (n = 15) muscles, CD20^+^ cells/mm^2^ (**d**) in healthy (n = 6), FSHD STIR− (n = 16) and FSHD STIR+ (n = 15) muscles and BDCA1^+^ cells/mm^2^ (**e**) in healthy (n = 6), FSHD STIR− (n = 17) and FSHD STIR+ (n = 15) muscles. The results are reported as mean ± SD. Groups were compared using Kruskal–Wallis test (§ < 0.05, §§ < 0.01, §§§*p* < 0.001) followed by Dunn’s multiple comparison test (*p < 0.05, **p < 0.01, ***p  < 0.001)

The presence of T and B lymphocytes and myeloid dendritic cells was assessed and compared among healthy, FSHD STIR− and FSHD STIR+ muscle sections by immunohistochemistry analysis (Fig. 2a). FSHD STIR+ muscles showed the highest count of CD4^+^ T lymphocytes (Fig. 2b), CD8^+^ T lymphocytes (Fig. 2c), CD20^+^ B lymphocytes (Fig. 2d) and BDCA1^+^ myeloid dendritic cells (Fig. 2e), compared to both FSHD STIR− and healthy muscles. FSHD STIR− muscles showed a slight not statistically significant increase in CD8^+^ T lymphocytes (Fig. 2c) and BDCA1^+^ myeloid dendritic cells (Fig. 2e) compared to healthy muscles. We then analyzed the immune cell infiltrates considering their localization between the muscle fibers (endomysial) or close to blood vessels (perivascular). CD4^+^ T lymphocytes were distributed equally between the endomysium and the vessels in healthy, FSHD STIR− and FSHD STIR+ muscles (Additional file 2: Fig. S1a). The number of CD8^+^ T lymphocytes was higher at the endomysium in all the muscles analyzed, compared with the perivascular site (Additional file 2: Fig. S1b). CD20^+^ B lymphocytes (Additional file 2: Fig. S1c) and BDCA1^+^ myeloid dendritic cells (Additional file 2: Fig. S1d) were found mostly at the endomysium in healthy and FSHD STIR− muscles while both cell populations were found equally distributed between the endomysium and perivascular regions in FSHD STIR+ muscles. To further analyze the differences in immune cells abundance in FSHD muscles, the total count of immune cells per sample was calculated as the sum of all immune cell infiltrates found in each sample (total immune cells/mm^2^). The mean ± 2SD total count of infiltrating immune cells in healthy muscles was used as the threshold (5.3 immune cells/mm^2^) to determine an upper control limit for muscle immune cell infiltrates. Over ninety percent (92.3%, 12/13) of FSHD STIR+ and 35.7% (5/14) of FSHD STIR− muscles showed an above threshold number of immune cells/mm^2^, and the former had a mean value of 3.9-fold higher compared to the latter (Table 2).

**Table 2:** Muscles with immune cell infiltrates over the upper normal limit threshold

Category of muscles	Muscles with immune cell infiltrates	% of muscles with immune cell infiltrates	Mean of immune cells/mm^2^ of muscles with immune cell infiltrates
Healthy	0/6	0.0	0.0
FSHD STIR−	5/14	35.7	12.9
FSHD STIR+	12/13	92.3	50.1

### Evaluation of muscle fibrosis in relation to immune cell infiltrates and MRI fat degeneration

**Fig. 3:**
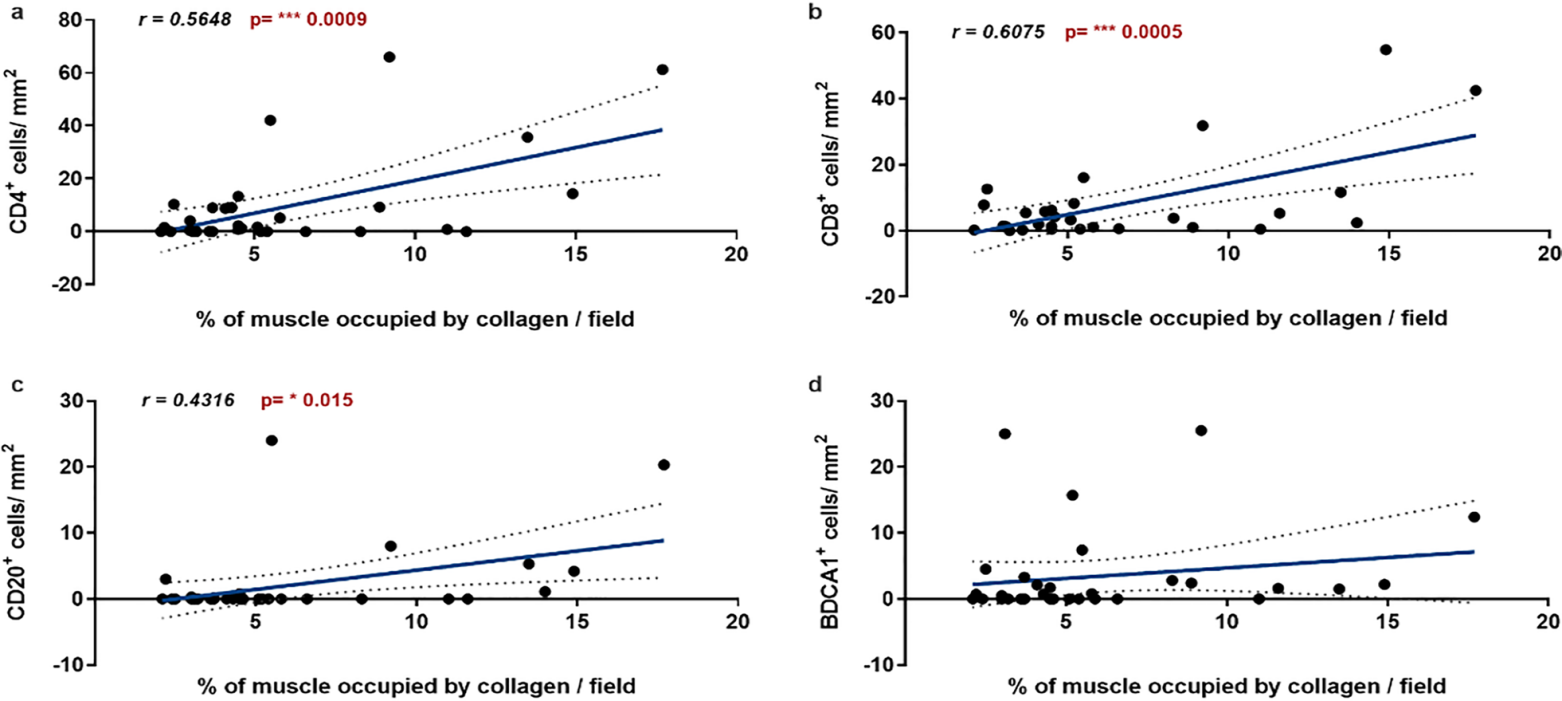
Muscle fibrosis and immune cell infiltration. Pearson correlations between the extent of fibrosis [fibrosis (%)/field] and the number of CD4^+^ (**a**), CD8^+^ (**b**), CD20^+^ (**c**) and BDCA1^+^ (**d**) cells in FSHD muscles. Pearson correlation coefficient (r value) and *p* value (*p*) are reported when statistically significant

**Fig. 4:**
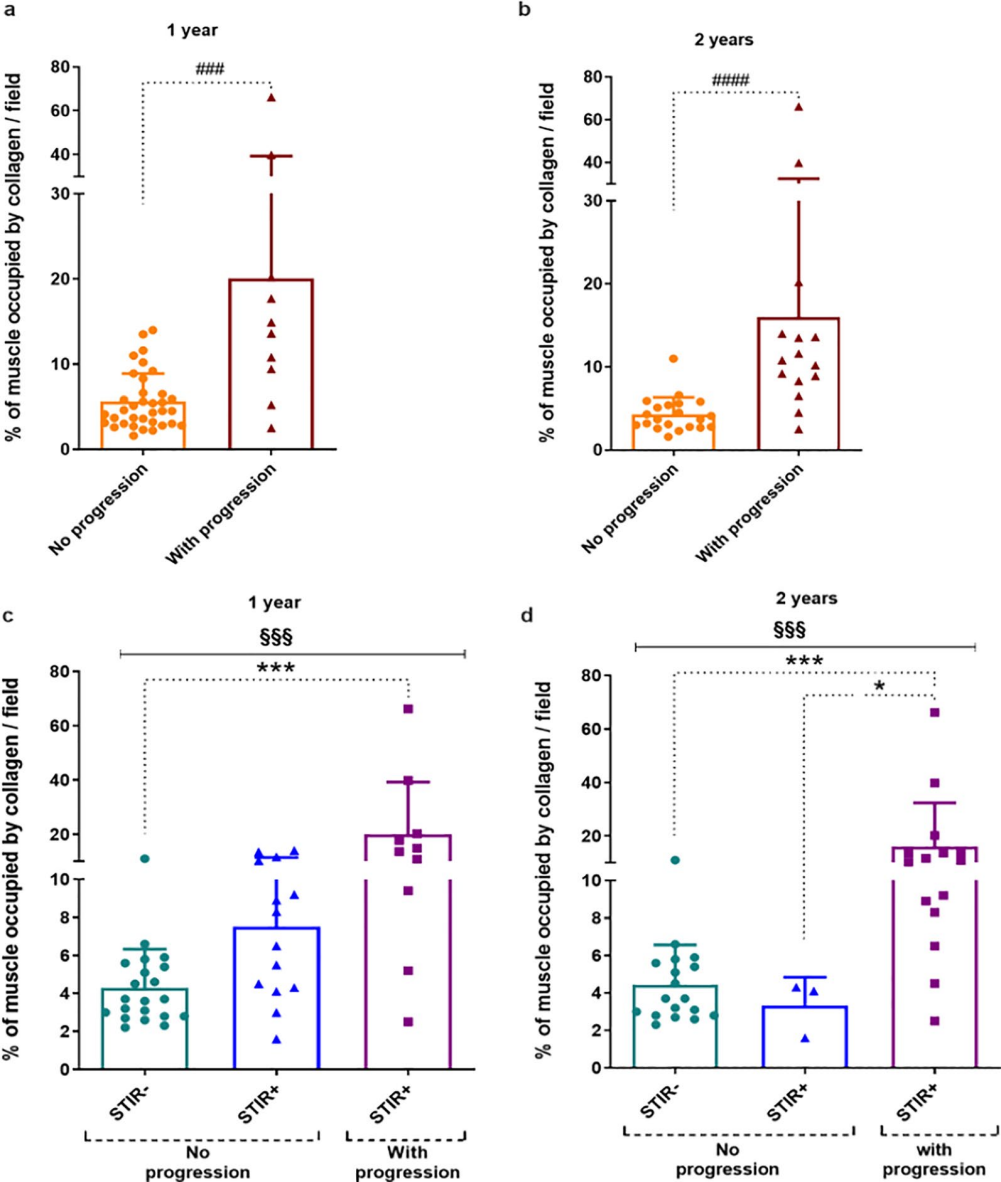
Collagen content and MRI fat degeneration. The graphs show the percentage of fibrosis in FSHD muscles without and with changes in fatty infiltration on T1w sequences in **a** 1-year MRI follow-up (no progression, n = 35; with progression, n = 10) and in **b** 2-year MRI follow-up (no progression, n = 21; with progression, n = 15). The graphs show the percentage of fibrosis in FSHD STIR− muscles without (n = 21), FSHD STIR+ muscles without (n = 14) and with (n = 10) changes in fatty infiltration on T1w sequences in **c** 1-year MRI follow-up and **d** 2-year MRI follow-up [FSHD STIR− muscles without (n = 18); FSHD STIR+ muscles without (n = 3); with (n = 15) progression]. The results are reported as mean ± SD. Comparison between two groups were done using Mann–Whitney test (^###^*p* < 0.001, ^####^*p* < 0.0001). Groups were compared using Kruskal–Wallis test (§§§*p* < 0.001) followed by Dunn’s test (*p < 0.05, ***p < 0.001)

We then evaluated the possible correlation between collagen deposition and immune cell infiltration in FSHD muscles. The correlation analysis showed that the amount of collagen significantly correlated with the number of CD4^+^ T lymphocytes (Fig. 3a), CD8^+^ T lymphocytes (Fig. 3b) and CD20^+^ B lymphocytes (Fig. 3c), but not with the number of BDCA1^+^ myeloid dendritic cells (Fig. 3d). To assess the relationship between muscle fibrosis and progression of fatty replacement, the sampled muscles were evaluated by MRI at the 1- and 2-year follow-up visits in 45 and 36 FSHD patients, respectively. FSHD muscles which showed a worsening in fatty infiltration at 1 year MRI follow-up had a collagen content at baseline of 3.6-fold significantly higher compared to FSHD muscles without T1 MRI signs of progression (Fig. 4a). Likewise, FSHD muscles with a worsening in fatty infiltration at 2 year MRI follow-up showed a collagen content at baseline of 3.7-fold significantly higher compared to FSHD muscles without sign of progression on T1w sequences (Fig. 4b). The 1-year MRI follow-up showed that 41.7% (10/24) FSHD STIR+ muscles and 0% (0/21) FSHD STIR− muscles exhibited progression of fatty infiltration on T1w sequences (Additional file 1: Table S1). FSHD STIR+ muscles with progression showed a significantly higher amount of collagen at baseline compared to FSHD STIR− muscles and a moderate albeit not significant increase compared to FSHD STIR+ muscles without progression (Fig. 4c). The 2-year MRI follow-up showed that 83.3% (15/18) FSHD STIR+ and 0% (0/18) FSHD STIR− muscles exhibited changes in fatty infiltration on T1w sequences (Additional file 1: Table S1). FSHD STIR+ muscles with progression showed a significantly higher content of collagen at baseline, compared to both FSHD STIR− and FSHD STIR+ muscles without progression (Fig. 4D). Notably, seven of nine (78%) of the STIR+ muscles without progression at 1 year which showed an increased fat content during the second year of MRI follow-up (Fig. 4d, Additional file 1: Table S1 samples FSHD#35, FSHD#37, FSHD#42, FSHD#44, FSHD#47, FSHD#51, FSHD#56), had the highest value of collagen at baseline (above the mean value of 8% of this group) (Fig. 4c, Additional file 1: Table S1).

### Immune cell infiltrates and fat degeneration in FSHD muscles

**Fig. 5:**
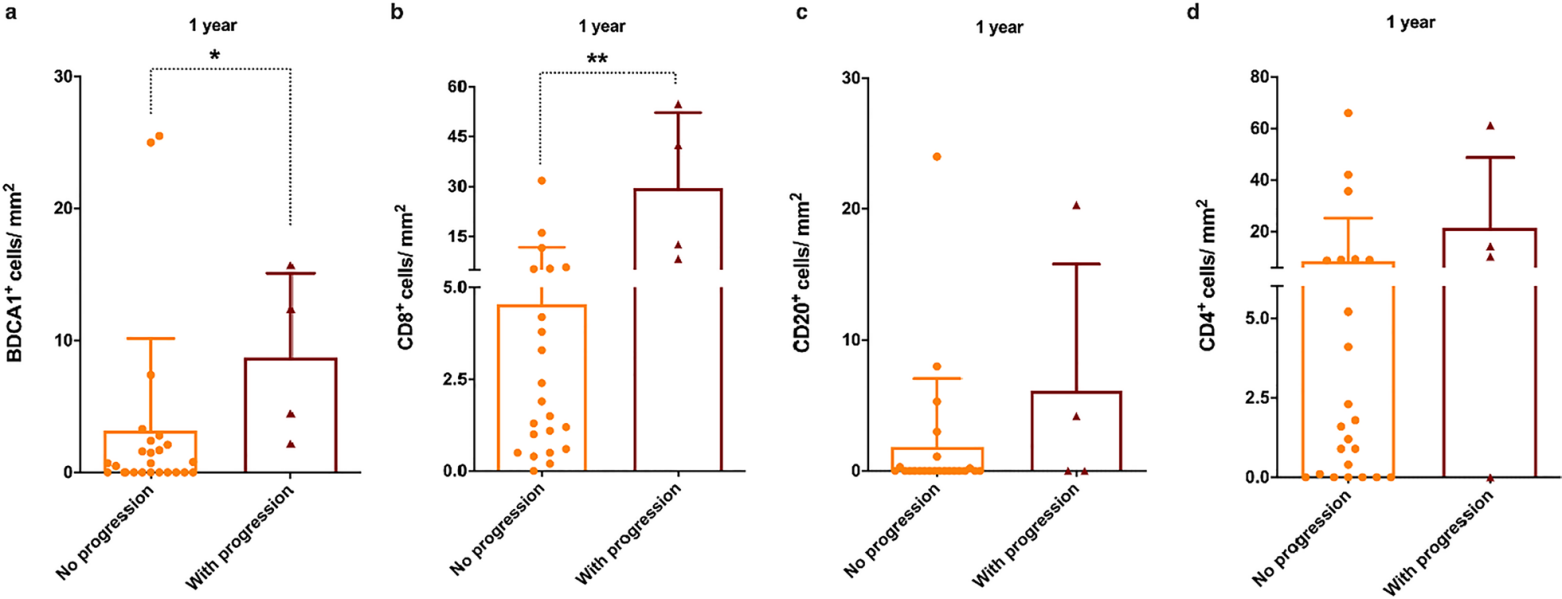
Immune infiltrates and fat degeneration in FSHD muscles at 1 year. The graphs show the **a** BDCA1^+^ (without progression n = 24, with progression n = 4), **b** CD8^+^ (without progression n = 22, with progression n = 4), **c** CD20^+^ (without progression n = 23, with progression n = 4) and **d** CD4^+^ (without progression n = 23, with progression n = 4) cells/mm^2^ in FSHD muscle sections without (orange framed bar) and with (burgundy framed bar) changes in fatty infiltration on T1w sequences at the 1-year MRI follow-up. The results are reported as mean ± SD and were compared using Mann–Whitney test (*p < 0.05 **p < 0.01)

**Fig. 6:**
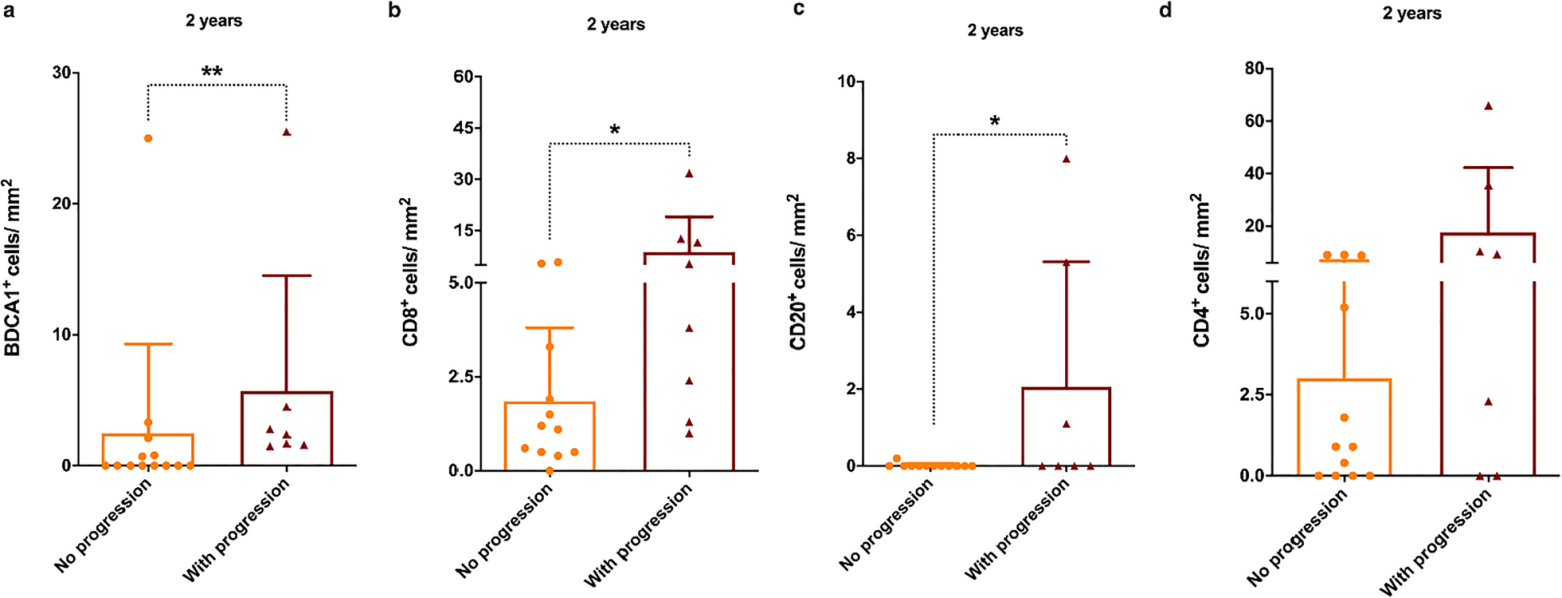
Immune infiltrates and fat degeneration in FSHD muscles at 2 years. The graphs show the **a** BDCA1^+^ (without progression n = 13, with progression n = 7), **b** CD8^+^ (without progression n = 12, with progression n = 8), **c** CD20^+^ (without progression n = 12, with progression n = 7) and **d** CD4^+^ (without progression n = 12, with progression n = 7) cells/mm^2^ in FSHD muscle sections without (orange framed bar) and with (burgundy framed bar) changes in fatty infiltration on T1w sequences at the 2-year MRI follow-up. The results are reported as mean ± SD and were compared using Mann–Whitney test (*p < 0.05 **p  < 0.01)

To investigate whether the presence of immune cell infiltrates correlated with the progression of muscle fat accumulation, we compared the number of T and B lymphocytes and myeloid dendritic cells between FSHD muscles with and without progression in fatty infiltration during the 1- and 2-year MRI follow-up. FSHD muscles with progression at the 1-year MRI follow-up showed a significantly high number of both BDCA1^+^ myeloid dendritic cells (Fig. 5a) and CD8^+^ T lymphocytes (Fig. 5b), but not of CD20^+^ B lymphocytes (Fig. 5c) and CD4^+^ T lymphocytes (Fig. 5d), compared to FSHD muscles without progression. FSHD muscles with progression at the 2-year MRI follow-up showed a significantly high number of BDCA1^+^ myeloid dendritic cells (Fig. 6a), CD8^+^ T lymphocytes (Fig. 6b) and CD20^+^ B lymphocytes (Fig. 6c), but not of CD4^+^ T lymphocytes (Fig. 6d), compared to FSHD muscles without progression.

## Discussion

Here we performed a retrospective cohort study focused on the evaluation of fibrosis in FSHD patients’ muscles with the final aim of identifying a candidate tissue biomarker with predictive value on muscle degeneration in FSHD patients. In recent years, MRI has turned out to be more sensitive than clinical tests in describing muscle involvement in muscular dystrophies, including FSHD, contributing to the comprehension of disease progression in single muscles [5–9, 11, 24]. However, differently from fatty replacement (a sign of terminal muscle degeneration determined by assessing the T1w sequences) and oedema/inflammation (a biomarker of disease activity in FSHD muscles with a prognostic value, evaluated by the presence of hyperintense signal on STIR-sequences), fibrosis, another relevant feature of muscle degeneration [28], cannot be determined by the commonly used MRI protocols. A link between the primary muscle disease and fibrosis was established years ago in muscular dystrophies [29] especially in Duchenne Muscular Dystrophy (DMD) [30]. Indeed, where fibrosis was found as the only myopathologic parameter significantly correlated with poor motor outcome, as evaluated in 10 year clinical follow-up study of DMD patients [31]. An impaired muscle regeneration potential is a complex process accountable for a dysregulation among myoblasts, satellite cells, non-satellite cells with myogenic potential, non-myogenic mesenchymal stromal cells and immune cells within the muscle niche [14–17]. In a FSHD mouse model, the inducible expression of *DUX4* caused a high pro-fibrotic state of muscles associated to an expansion of fibro-adipogenic progenitors and an increased inflammatory response [22, 32]. Accordingly, we have recently demonstrated a positive correlation between the expansion of non-myogenic mesenchymal cells and the amount of fibrosis in FSHD patients’ muscles [21] although an association of this finding with an altered *DUX4* signature has not been clarified yet. Until now, however, the correlation between the disease progression (in terms of MRI fatty infiltration), collagen deposition (as an indicator of fibrosis), and immune cell infiltration has not been established for FSHD patients. In this study, we histologically determined the extent of fibrosis in a large cohort of FSHD muscles by evaluating their collagen content. Our data showed that 85.2% of FSHD STIR+ and 42.9% of FSHD STIR− muscles had higher collagen deposition compared to healthy muscles. Moreover, the evaluation of fibrosis in relation to MRI progression of fatty infiltration at the 1- and 2-year follow-up showed that the extent of collagen positively correlated with fatty replacement of FSHD muscles. These results may suggest that, as observed in other muscular dystrophies [29–31], an excessive collagen deposition is a primary event that precedes muscle degeneration also for the FSHD. Moreover, fibrosis proved to be an independent prognostic biomarker of muscle degeneration in FSHD because it was able to distinguish between progressive and not progressive FSHD STIR+ muscles in a timeframe of 2 years. A limitation to this study could be the different numbers of the two categories of muscles analyzed [not progressive STIR+ muscles (n = 3) versus progressive STIR+ muscles (n = 15), respectively], although they significantly differ in the extent of fibrosis. There are numerous publications supporting the interdependence between inflammatory response and fibrosis [33, 34], even in muscular dystrophies [35], although it has to be deepened for FSHD. We evaluated immune infiltration in FSHD muscles and consistently with previous reports [3, 13, 18, 19], we found that the most prevalent immune cell infiltrates in our cohort of FSHD muscles were T lymphocytes (both CD4^+^ and CD8^+^), and the FSHD STIR+ muscles presented the highest amount of immune cells infiltrating the tissue. We observed a low-grade of immune cell recruitment in 5/14 FSHD STIR− muscles. The total immune cells found in the 5 FSHD STIR− muscles differed to the ones observed in FSHD STIR+ muscles (50.1 immune cells/mm^2^ compared to 12.9 immune cells/mm^2^, respectively) and in spite of this low-grade immune cell recruitment, the 5 FSHD STIR− muscles did not show MRI signs of disease progression in the 2-year timeframe, as the other FSHD STIR− muscles analyzed. In line with these findings, a recent RNA-sequencing study demonstrated the upregulation of inflammatory genes in both STIR+ and STIR− FSHD muscle biopsies, even in the absence of *DUX4* and its target genes expression [12]. In the light of different studies supporting the causative role of inflammation in driving fibrosis in skeletal muscle [28, 36, 37], we evaluated a possible correlation among the immune cells found in FSHD muscles and the amount of fibrosis. Although the specific interrelation between immune cells and fibrosis in FSHD remains to be investigated, we observed a positive correlation between fibrosis and the presence of T and B lymphocytes, suggesting a possible specific role of these infiltrates in stimulating the accumulation of collagen in FSHD muscles like described in DMD [35, 38]. However, the observed inflammatory patterns in FSHD share similarities with other muscular dystrophies [35, 39–42] raising the possibility that a mechanism similar to what is seen in inflammatory idiopathic myopathies could also be occurring in FSHD. In the context of B lymphocytes, although there is no direct evidence regarding their action in FSHD, infiltrating plasma cells have been observed in patients with polymyositis [43]. Furthermore previous studies have reported in FSHD the existence of sarcolemmal complement deposits in non-necrotic muscle fibers [44], an increased expression of complement system proteins [10] and elevated levels of circulating inflammatory cytokines [19] but we did not extensively investigate the presence of specific inflammatory subsets or the activation of the complement system because our interest focused on investigating the immune cell infiltration in relation to fibrosis.

In conclusion, this study introduces a promising new biomarker in FSHD, besides MRI-derived parameters [45]. Here we show that muscle fibrosis, a parameter undetectable by qualitative MRI, has a predictive value towards muscle degeneration in FSHD muscles, regardless of the STIR signal. Although current clinical trials for FSHD utilize skeletal muscle biopsies to evaluate therapy efficacy [46], we hope our study will increase the interest in fibrosis in FSHD. This, in turn, may accelerate the development and the adoption of strategies to non-invasively detect and quantify fibrosis in FSHD patients.

### Supplementary Information

**Additional file 1: Table S1.** Informative data of healthy volunteers, FSHD patients and muscle biopsies. M: male, F: female, CSS: clinical severity score, /: not assessed. Changes in T1w sequences were assessed at 1- and 2-year MRI follow-up. NO means no changes, YES means an increase in fatty replacement on T1 MRI signal. Immune cell infiltrates (CD4, CD8, CD20, BDCA1) and total count of immune cell infiltrates reported as positive cells/mm^2^.

**Additional file 2: Fig. S1.** Evaluation of endomysial and perivascular immune infiltrates in healthy and FSHD muscles: The graphs show the endomysial and perivascular distribution of CD4^+^ cells/mm^2^ (**A**) in healthy (n = 6), FSHD STIR− (n = 16) and FSHD STIR+ (n = 15) muscles, CD8^+^ cells/mm^2^ (**B**) in healthy (n = 6), FSHD STIR− (n = 16) and FSHD STIR+ (n = 15) muscles, CD20^+^ cells/mm^2^ (**C**) in healthy (n = 6), FSHD STIR− (n = 14) and FSHD STIR+ (n = 15) muscles and BDCA1^+^ cells/mm^2^ (**D**) in healthy (n = 6), FSHD STIR− (n = 17) and FSHD STIR+ (n = 15) muscles. The results are reported as mean ± SD. Groups were compared using Kruskal–Wallis test (§ <0.05, §§ <0.01, §§§*p* < 0.001,) followed by Dunn’s multiple comparison test (*p < 0.05, **p < 0.01). Comparison between two groups were done using Mann-Whitney test (^#^*p* < 0.05, ^##^*p* < 0.01).

## Data Availability

All data generated or analysed during this study are included in this published article [and its Additional files].
