# Supplementary material for: Muscle fibrosis as a prognostic biomarker in facioscapulohumeral muscular dystrophy: a retrospective cohort study
Source: Acta Neuropathol Commun. 2023 Oct 17;11:165. doi: 10.1186/s40478-023-01660-4 (PMC10583430; doi:10.1186/s40478-023-01660-4)
Supplement: Supplementary file 2 — Additional file 2: Fig. S1. Evaluation of endomysial and perivascular immune infiltrates in healthy and FSHD muscles: The graphs show the endomysial and perivascular distribution of CD4+ cells/mm2 (A) in healthy (n = 6), FSHD STIR− (n = 16) and FSHD STIR+ (n = 15) muscles, CD8+ cells/mm2 (B) in healthy (n = 6), FSHD STIR− (n = 16) and FSHD STIR+ (n = 15) muscles, CD20+ cells/mm2 (C) in healthy (n = 6), FSHD STIR− (n = 14) and FSHD STIR+ (n = 15) muscles and BDCA1+ cells/mm2 (D) in healthy (n = 6), FSHD STIR− (n = 17) and FSHD STIR+ (n = 15) muscles. The results are reported as mean ± SD. Groups were compared using Kruskal–Wallis test (§ <0.05, §§ <0.01, §§§p < 0.001,) followed by Dunn’s multiple comparison test (*p < 0.05, **p < 0.01). Comparison between two groups were done using Mann-Whitney test (#p < 0.05, ##p < 0.01). [file 40478_2023_1660_MOESM2_ESM.pdf]

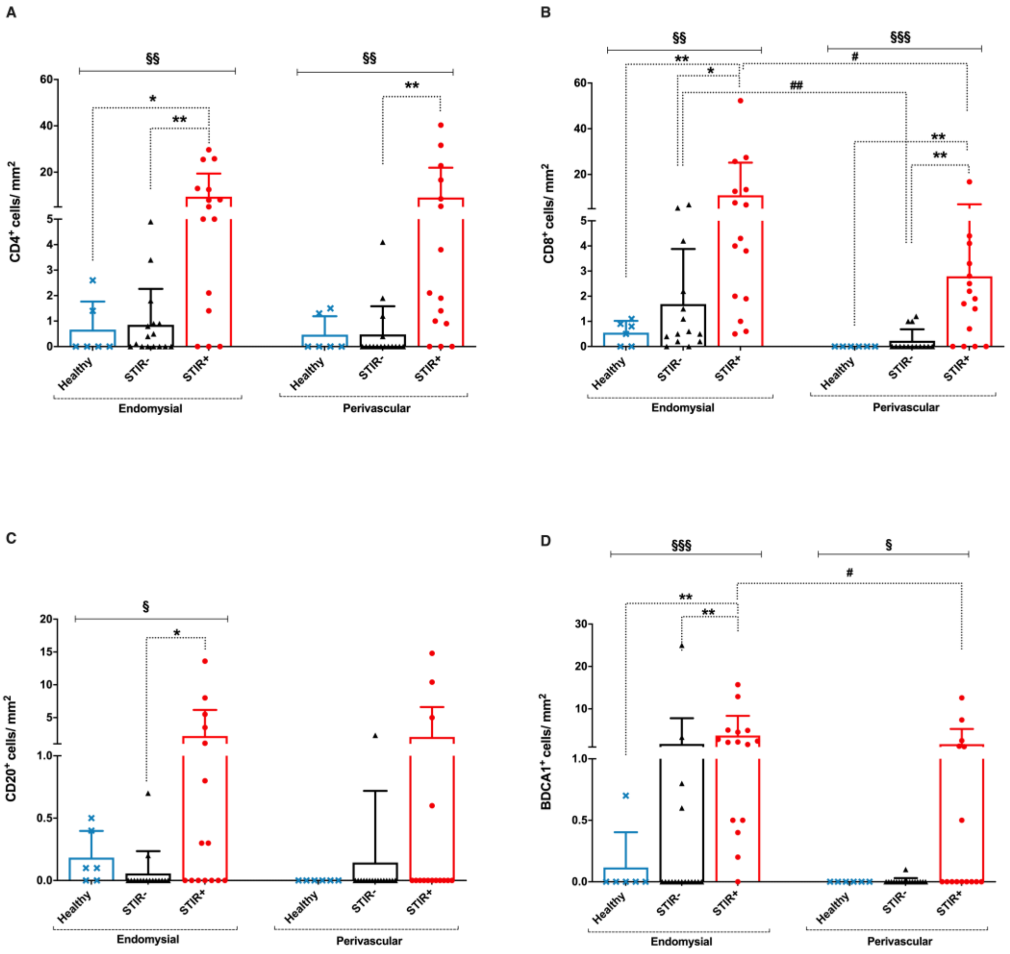

Supplemental Figure 1. Evaluation of endomysial and perivascular immune infiltrates in healthy and FSHD muscles: The graphs show the endomysial and perivascular distribution of $CD4^+$ cells/mm^2 (**A**) in healthy (n=6), FSHD STIR- (n=16) and FSHD STIR+ (n=15) muscles, $CD8^+$ cells/mm^2 (**B**) in healthy (n=6), FSHD STIR- (n=16) and FSHD STIR+ (n=15) muscles, $CD20^+$ cells/mm^2 (**C**) in healthy (n=6), FSHD STIR- (n=14) and FSHD STIR+ (n=15) muscles and $BDCA1^+$ cells/mm^2 (**D**) in healthy (n=6), FSHD STIR- (n=17) and FSHD STIR+ (n=15) muscles. The results are reported as mean ± SD. Groups were compared using Kruskal-Wallis test (§ <0.05, §§ <0.01, §§§p < 0.001,) followed by Dunn's multiple comparison test (*p <0.05, **p <0.01). Comparison between two groups were done using Mann-Whitney test ($^{\#}$p <0.05, $^{\#\#}$p <0.01).
